# New Insights into the Function of the Immunoproteasome in Immune and Nonimmune Cells

**DOI:** 10.1155/2015/541984

**Published:** 2015-10-08

**Authors:** Hiroaki Kimura, Patrizio Caturegli, Masafumi Takahashi, Koichi Suzuki

**Affiliations:** ^1^Division of Inflammation Research, Center for Molecular Medicine, Jichi Medical University School of Medicine, 3311-1 Yakushiji, Shimotsuke, Tochigi 329-0498, Japan; ^2^Department of Pathology, The Johns Hopkins School of Medicine, Ross 656, 720 Rutland Avenue, Baltimore, MD 20205, USA; ^3^Department of Clinical Laboratory Science, Faculty of Medical Science, Teikyo University, 2-11-1 Kaga, Itabashi, Tokyo 173-8605, Japan

## Abstract

The immunoproteasome is a highly efficient proteolytic machinery derived from the constitutive proteasome and is abundantly expressed in immune cells. The immunoproteasome plays a critical role in the immune system because it degrades intracellular proteins, for example, those of viral origin, into small proteins. They are further digested into short peptides to be presented by major histocompatibility complex (MHC) class I molecules. In addition, the immunoproteasome influences inflammatory disease pathogenesis through its ability to regulate T cell polarization. The immunoproteasome is also expressed in nonimmune cell types during inflammation or neoplastic transformation, supporting a role in the pathogenesis of autoimmune diseases and neoplasms. Following the success of inhibitors of the constitutive proteasome, which is now an established treatment modality for multiple myeloma, compounds that selectively inhibit the immunoproteasome are currently under active investigation. This paper will review the functions of the immunoproteasome, highlighting areas where novel pharmacological treatments that regulate immunoproteasome activity could be developed.

## 1. Introduction

The immunoproteasome is a large proteolytic machine derived from the constitutive proteasome [1, 2] and plays a critical role in homeostasis and immunity. The constitutive proteasome is expressed ubiquitously in the body, where it degrades ubiquitinated proteins including transcriptional factors and proteins required for cell cycle progression [3, 4]. Since the primary role of the immunoproteasome is to process antigens for presentation on major histocompatibility complex (MHC) class I molecules to CD8^+^ T lymphocytes [5], the immunoproteasome degrades various proteins, including viral proteins. Therefore, the immunoproteasome plays an important role during viral infection [6, 7]. The expression of the immunoproteasome is induced by interferon-*γ* (IFN-*γ*) and tumor necrosis factor-*α* (TNF-*α*) [8] under inflammatory conditions, such as infections, and autoimmune diseases when inflammatory cytokines are present [9]. Accordingly the immunoproteasome is controlled by factors that impact the immune system [10–13]. Interestingly, various roles for the immunoproteasome in nonimmune cells have been reported recently [14–16], suggesting that there could still be unknown roles for the immunoproteasome.

This review summarizes the roles of the immunoproteasome and recent efforts to develop novel therapeutic approaches by regulating immunoproteasome activity.

## 2. Structure and Activity of the Immunoproteasome

**Figure 1:**
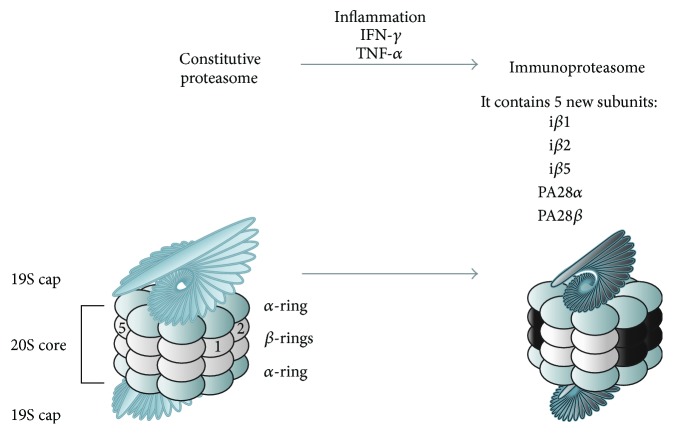
Structure of the constitutive proteasome and the immunoproteasome. The constitutive proteasome is composed of two pairs of inner *β*-rings, two pairs of outer *α*-rings, and two caps (19S regulatory complexes). Inflammatory cytokines induce the expression of the five subunits (i*β*1 [LMP2], i*β*2 [LMP10], i*β*5 [LMP7], PA28*α*, and PA28*β*), which assemble on the proteasome core to create the immunoproteasome. When the induced subunits replace the *β* subunits and 19S regulatory complex, the resulting multiprotein complex is called the immunoproteasome.

The immunoproteasome is a large proteolytic machinery derived from the constitutive proteasome (also known as the 26S proteasome) and is expressed abundantly in immune cells, such as antigen-presenting cells [17–19]. The constitutive proteasome is expressed in the cytosol and nucleus of most cells, where it degrades ubiquitinated proteins to maintain cell viability and homeostasis [4, 20]. For example, the constitutive proteasome degrades long-lived proteins, including proteins used for cell cycle progression and gene transcription. It is a large barrel-shaped protein complex [21, 22] composed of a catalytic 20S core proteasome and two 19S regulatory complex components located at both ends of the 20S core proteasome (Figure 1, left panel). The 20S core proteasome has two pairs of outer *α* rings consisting of seven *α* subunits and two pairs of inner *β* rings consisting of seven *β* subunits. The three *β* subunits (*β*1, *β*2, and *β*5) have proteolytic activities, including caspase-like activity for *β*1, trypsin-like activity for *β*2, and chymotrypsin-like activity for *β*5 [23]. The 20S core proteasome is usually capped at both ends by the 19S regulatory complex [21, 22]. The 19S regulatory complex recognizes ubiquitinated proteins and transfers them into the core of the proteasome where they are degraded by proteolysis.

When a cell is exposed to inflammatory stimuli, such as IFN-*γ* and TNF-*α*, five of the proteasome subunits are substituted with more efficient subunits: *β*1 is replaced with i*β*1 (also known as large multifunctional peptidase 2 (LMP2) or proteasome subunit beta type 9 (PSMB9)), *β*2 is replaced with i*β*2 (also known as LMP10, multicatalytic endopeptidase complex-like-1 (MECL-1), or PSMB10), *β*5 is replaced with i*β*5 (also known as LMP7 or PSMB8), and the 19S regulatory complex is replaced with the 11S regulator composed of Proteasome Activator *α* (PA28*α*) and PA28*β* (Figure 1, right panel and Table 1) [24–29]. This modified proteasome is called the immunoproteasome and it performs its proteolytic functions more efficiently than the constitutive proteasome [1]. For example, it degrades viral proteins for antigen presentation [7] and also processes ubiquitinated proteins, as does the constitutive proteasome [30]. Expression of the immunoproteasome subunits can be induced in nonimmune cells stimulated by IFN-*γ* [13, 16, 31]. Therefore, the immunoproteasome plays multiple roles, and the function of the immunoproteasome is not restricted to the immune system.

## 3. Roles of the Immunoproteasome during Infection

**Figure 2:**
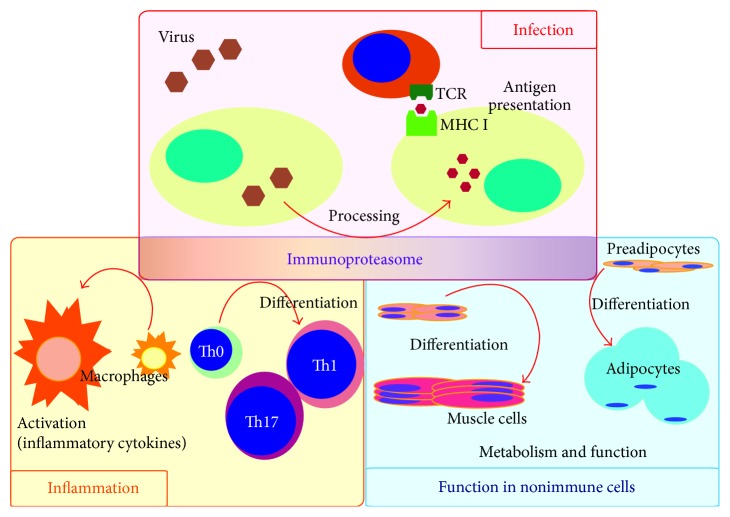
The immunoproteasome as a potential therapeutic target. The immunoproteasome plays an important role in immune responses, including processing viral proteins for antigen presentation, T cell differentiation, and macrophage activation. Recent studies have identified that the immunoproteasome is present in nonimmune cells, where it regulates cell differentiation and function.

The best characterized role of the immunoproteasome is the processing of proteins in order to present antigenic peptides on MHC class I molecules (Figure 2) [32]. Deficiency of the immunoproteasome in mice reduces CD8^+^ T cell activation in hepatitis B virus (HBV) infection, lymphocytic choriomeningitis virus (LCMV) infection, and influenza virus infection [7, 33, 34], although not in coxsackie virus B3 (CVB3) infection [35]. The immunoproteasome is also important for activating the immune system against viral infection. For example, LMP2 deficiency reduced inflammatory cytokine (IL-1*β*, IL-6, and TNF-*α*) production during influenza viral infection [36]. Inflammatory cytokines, such as type I and type II IFNs and TNF-*α*, induce the expression of the subunits that assemble into the immunoproteasome [37, 38]. Hepatitis C viral (HCV) infection or poly(I : C)-stimulation (mimicking viral infection) induces the expression of type I IFN (IFN-*β*) and the immunoproteasome subunits in hepatocytes [38]. Suppression of IFN-*β* inhibits expression of the immunoproteasome, and type I IFN (IFN-*α*) treatment induces immunoproteasome expression in hepatocytes. Furthermore, Keller et al. showed that murine gammaherpesvirus-68 (MHV-68) infection induced expression of the immunoproteasome subunits in alveolar macrophages in the lung [16]. Thus, viral infection, IFN production, and expression of the immunoproteasome are strongly linked.

It should be noted that immunity against viral infections is not completely dependent on the immunoproteasome because there are some antiviral immune responses independent of the immunoproteasome [39]. In fact, mice lacking all of the immunoproteasome activities, generated by treating LMP2/LMP10 double-deficient mice with a LMP7-selective inhibitor, were still able to induce IFN-*γ*-producing cytotoxic CD8^+^ T cells upon LCMV infection [39].

## 4. Roles of the Immunoproteasome in Inflammatory Diseases

The immunoproteasome is involved in the pathogenesis of numerous inflammatory diseases, such as autoimmune diseases, by influencing T cell polarization, signaling through the nuclear factor-*κ*B (NF-*κ*B) pathway, and the production of inflammatory cytokines by macrophage [40–45]. For example, Kalim et al. reported that LMP7 deficiency suppressed the differentiation of naïve CD4^+^ T cells to Th1 and Th17 cells and instead promoted their differentiation to regulatory T cells (Figure 2) [46]. Maldonado et al. reported that deficiency of the immunoproteasome influenced NF-*κ*B signaling [47]. The constitutive proteasome is involved in NF-*κ*B signaling by degrading ubiquitinated I-*κ*B. It remains to be defined how the constitutive proteasome and the immunoproteasome regulate NF-*κ*B. Reis et al. reported that upregulated LMP7 expression in mouse macrophages due to LPS stimulation was suppressed by treatment with immunoproteasome inhibitors, including an LMP7 inhibitor (Figure 2) [48].

The immunoproteasome is essential for processing antigenic epitopes that are presented on MHC class I molecules to activate CD8^+^ T lymphocytes. The immunoproteasome is also involved in the regulation of NF-*κ*B, which is essential for the transcription of many genes that encode inflammatory cytokines. Therefore, the activity of the immunoproteasome is essential in various inflammatory scenarios that result in pathological conditions. Thus, attempts were made to inhibit the immunoproteasome to identify potential treatments for inflammatory diseases. ONX-0914 (also known as PR-957) is a selective LMP7 inhibitor, which has been used as a treatment for autoimmune diseases in animal models. Muchamuel et al. reported that ONX-0914 attenuated experimental arthritis by blocking inflammatory cytokine expression [10]. As we mentioned, this LMP7 inhibitor blocked antigen presentation by MHC class I, suppressed the proliferation and activation of CD8^+^ T cells and Th17 cells, and lowered the production of inflammatory cytokines. The inhibitory effects probably contribute to the attenuation of disease progression in experimental arthritis.

Basler et al. showed that treatment with ONX-0914 significantly attenuated the clinical symptoms of experimental colitis and encephalomyelitis in mice [11, 12]. Expression of the immunoproteasome subunits (LMP2, LMP7, and LMP10) was upregulated in colitis lesions, which was induced in mice deficient in each of the immunoproteasome subunits. Colon lesions were significantly ameliorated in each of the deficient mouse strains compared to wild-type controls, and the amelioration was associated with suppressed inflammatory cytokine expression (TNF-*α*, IL-1*β*, IFN-*γ*, IL-6, IL-23, and IL17). Then, they examined the effect of ONX-0914 in experimental colitis and showed that treatment with ONX-0914 significantly improved colitis lesions. Although deficiency of the individual immunoproteasome subunits (i.e., LMP2, LMP7, or MECL-1) did not improve disease in a mouse model of experimental encephalomyelitis, treatment with ONX-0914 significantly attenuated disease progression and prevented a second exacerbation [12]. The authors mentioned that this discrepancy between immunoproteasome subunit-deficient mice and inhibitor-treated mice could be explained by the fact that endogenous chymotrypsin-like activity in monocytic cells contributes to pathogenesis and ONX-0914 inhibits chymotrypsin-like activity [12]. Deficiency of a single subunit is not able to suppress all chymotrypsin-like activity in the immunoproteasome because both LMP2 and LMP7 have chymotrypsin-like activity [49, 50]. Overall, these studies suggest that ONX-0914 has potential for treating autoimmune diseases.

The immunoproteasome is involved in the pathogenesis of chronic thyroiditis [13]. Transgenic mice that express IFN-*γ* specifically in the thyroid develop chronic thyroiditis and hypothyroidism [51, 52]. In this mouse model, LMP2 deficiency significantly improved inflammatory thyroid morphology and function [13]. Nagayama et al. reported that treatment with ONX-0914 improved Th1-type autoimmune thyroid disease (Hashimoto's thyroiditis), but not Th2-type autoimmune thyroid disease (Graves' disease), using mouse models [53]. Treatment with ONX-0914 suppressed IFN-*γ* and IL-17 expression in the thyroid, which supports Basler's results.

LMP7 deficiency or treatment with ONX-0914 (a selective inhibitor of LMP7) seems to suppress inflammatory diseases with Th1 and Th17 cell-mediated inflammation. One report showed that LMP7 deficiency reduced Th2 responses in an asthma model [54]. LMP7 deficiency suppressed expression of the Th2 cytokines IL-4, IL-5, and IL-13 and infiltration of immune cells into the lung. The detailed mechanism of how LMP7 deficiency influences T cell polarization is still undefined. Because either Th1 or Th2 polarization is normally involved in the pathogenesis of many inflammatory diseases, it is necessary to know how the immunoproteasome influences T cell polarization in various inflammatory disease contexts in order to translate these findings to clinical studies.

## 5. Roles of the Immunoproteasome in Nonimmune Cells

Recent studies have examined the role of the immunoproteasome in nonimmune cells. Cui et al. reported that the immunoproteasome regulated skeletal muscle differentiation (Figure 2) [14]. They found that inhibiting the immunoproteasome by short hairpin RNA suppressed muscle differentiation using the mouse myoblast cell line C2C12 and human skeletal muscle myoblasts. Proapoptotic proteins and apoptotic cells were upregulated by the treatment, which indicates that the immunoproteasome also regulates the degradation of proteins associated with apoptosis, as does the constitutive proteasome. They speculated that the immunoproteasome influences transcriptional factors associating with muscle differentiation. Zu et al. reported that the immunoproteasome regulated cardiac muscle mass in diabetic mice [55]. Streptozotocin (STZ) is commonly used to induce diabetic conditions in the experimental animal model. They showed that LMP2 expression was decreased in the hearts of STZ-injected mice. On the other hand, the expression of phosphatase and tensin homologue deleted on chromosome ten (PTEN) was upregulated, which impaired muscle regeneration [56]. LMP2 deficiency itself also leads to loss of cardiac muscle mass, which decreased cardiac function [55].

LMP7 has been associated with human disease, although no association has been found with the other immunoproteasome subunits (LMP2, LMP10, PA28*α*, and PA28*β*). LMP7 mutation causes disease with autoinflammation and lipodystrophy [15, 57–59], and the number of cases is increasing [60, 61]. As we described above, LMP7 plays a critical role in the immune system and is involved in cytokine expression. LMP7 mutation in humans causes abnormalities in cytokine expression, as listed in Table 2. Kitamura et al. showed that IL-6 expression was significantly higher in the skin lesions or sera of patients with LMP7 mutation [15], similar to other reports [57, 60]. In particular, an association of LMP7 and lipodystrophy is interesting. Reduction of LMP7 expression by siRNA suppressed adipogenesis in 3T3-L1 cells (Figure 2) [15]. LMP7 might be involved in lipid metabolic disorders because LMP7 is also associated with insulin-dependent diabetes mellitus [62], and inflammation is involved in the pathophysiology of metabolic diseases [63, 64]. To date, the role of the immunoproteasome in metabolic disorders and the endocrine system is poorly understood. We showed previously that overexpression of LMP2 was involved in the pathogenesis of chronic thyroid inflammation and hypothyroidism as described above [13]. In that study, we found that LMP2 was expressed in oxyphilic thyrocytes in humans and mice, and deletion of LMP2 in mice dramatically improved thyroid function and thyrocyte morphology [13]. These findings suggest an association between the immunoproteasome and endocrine metabolic function.

The lung is a vulnerable site for pathogens that induce chronic inflammation. Therefore, the immunoproteasome may play an important role in the lung. In fact, Keller et al. reported that immunoproteasome expression was detected in the lung parenchymal cells, for example, alveolar type I and II cells, fibroblasts, and bronchial epithelial cells at basal levels [16]. Viral infection and subsequent IFN secretion upregulated immunoproteasome expression in the lung. It is still not clear why those cells in the lung constitutively express LMP7 without infection or inflammation.

Considering the involvement of the immunoproteasome in cell differentiation and function, the immunoproteasome is important in nonimmune cells, too. Expression of the immunoproteasome in nonimmune cells during normal conditions has been found, although its role is not fully understood. Therefore, the role of the immunoproteasome in nonimmune cells should be addressed using mice deficient in the various immunoproteasome subunits, by knockdown of the immunoproteasome genes and with immunoproteasome inhibitors.

## 6. Immunoproteasome Inhibitors and Their Clinical Relevance for Inflammatory Diseases and Neoplasms

ONX-0914 is, thus far, the best characterized immunoproteasome inhibitor. As shown in the previous section, ONX-0914 specifically inhibits LMP7 (i*β*5), and it has been used in animal models and* in vitro* studies of inflammatory diseases [10–12, 46, 53, 65]. Although selective inhibitors for LMP2 were not available when we reported that LMP2 deficiency suppressed thyroid inflammation and improved thyroid function [13], we expect that such inhibitors will be used to treat patients with chronic thyroiditis in the future. More studies are needed to analyze the mechanisms underlying the action of LMP2 on thyroid function.

Recently, immunoproteasome inhibitors have been investigated for application in clinical settings to treat hematopoietic neoplasms. Bortezomib is an inhibitor of *β*5, a component of the constitutive proteasome, and has been used to treat multiple myeloma and mantle cell lymphoma [66]. Since the proteasome is responsible for the degradation of proteins involved in cell cycle progression, inhibition of proteasome function by bortezomib results in an accumulation of undigested proteins that leads to cell death.

Alternative treatments that overcome bortezomib-resistant malignancies have been characterized [66]. ONX-0912 is an inhibitor of both LMP7 (i*β*5) and *β*5 and is effective for bortezomib-resistant myelomas [66, 67]. UK-101 and IPSI-001 selectively inhibit LMP2 and exhibit antitumor activity against malignant myelomas [68, 69]. Carfilzomib is effective for the treatment of myelomas and small cell lung cancers [70, 71]. Proteasome subunits LMP7 (i*β*5), LMP2 (i*β*1), and *β*5 have chymotrypsin-like activity. Since carfilzomib is a potent inhibitor of chymotrypsin-like activity [70], it appears likely that chymotrypsin-like activity is important for maintaining the proliferation of hematologic tumor cells. Precise differences in the chymotrypsin-like activity among the three subunits should be defined in order to understand how malignant cells acquire resistance to those proteasome inhibitors.

## 7. Conclusion

Regulating immunoproteasome expression and activity is a powerful tool for controlling cell function, which includes cell metabolism, differentiation, and immune regulation. So far, inhibitors of the immunoproteasome are widely available and applicable to the treatment of many inflammatory diseases and hematopoietic malignancies. In the near future, colitis and rheumatoid arthritis could be candidates for developing new treatments that target the immunoproteasome. In addition, metabolic diseases could provide additional candidates because the immunoproteasome is involved in both adipogenesis and inflammation of adipose tissue. As described in this review, most basic studies on the roles of the immunoproteasome in disease models have been achieved using mice (summarized in Figure 2). Since immunoproteasome enzymatic activity differs between species [72], findings from such basic studies should be carefully interpreted when considering the development of new therapeutic applications.

## Figures and Tables

**Table 1:** Human immunoproteasome subunits.

Subunit	Proteolytic activity	Molecular weight	Chromosome	Alternative name
i*β*1	Chymotrypsin-like	23.3 kD	6p21.3	PSMB9, LMP2
i*β*2	Undefined	28.9 kD	16q22.1	PSMB10, LMP10, and MECL-1
i*β*5	Chymotrypsin-like	30.4 kD	6p21.3	PSMB8, LMP7
PA28*α*	N/A	28.7 kD	14q11.2	
PA28*β*	N/A	27.4 kD	14q11.2	

**Table 2:** Human PSMB8 (LMP7 gene) alleles.

Mutation	Influenced cytokines	Reference
Thr 75 Met	IL-6, IL-8, and IFN-*γ*	[58–60]
Cys 135 termination	Unknown	[60]
Gly 197 Val	IL-6	[15]
Gly 201 Val	IL-6, IL-10	[57]
